# Differential Effects of Aging on Fore– and Hindpaw Maps of Rat Somatosensory Cortex

**DOI:** 10.1371/journal.pone.0003399

**Published:** 2008-10-14

**Authors:** Marianne David-Jürgens, Lydia Churs, Thomas Berkefeld, Roberto F. Zepka, Hubert R. Dinse

**Affiliations:** Institute for Neuroinformatics, Department of Theoretical Biology, Neural Plasticity Lab, Ruhr-University Bochum, Bochum, Germany; University of Sydney, Australia

## Abstract

Getting older is associated with a decline of cognitive and sensorimotor abilities, but it remains elusive whether age-related changes are due to accumulating degenerational processes, rendering them largely irreversible, or whether they reflect plastic, adaptational and presumably compensatory changes. Using aged rats as a model we studied how aging affects neural processing in somatosensory cortex. By multi-unit recordings in the fore- and hindpaw cortical maps we compared the effects of aging on receptive field size and response latencies. While in aged animals response latencies of neurons of both cortical representations were lengthened by approximately the same amount, only RFs of hindpaw neurons showed severe expansion with only little changes of forepaw RFs. To obtain insight into parallel changes of walking behavior, we recorded footprints in young and old animals which revealed a general age-related impairment of walking. In addition we found evidence for a limb-specific deterioration of the hindlimbs that was not observed in the forelimbs. Our results show that age-related changes of somatosensory cortical neurons display a complex pattern of regional specificity and parameter-dependence indicating that aging acts rather selectively on cortical processing of sensory information. The fact that RFs of the fore- and hindpaws do not co-vary in aged animals argues against degenerational processes on a global scale. We therefore conclude that age-related alterations are composed of plastic-adaptive alterations in response to modified use and degenerational changes developing with age. As a consequence, age-related changes need not be irreversible but can be subject to amelioration through training and stimulation.

## Introduction

Getting older is associated with a decline of cognitive and sensorimotor abilities. An increased life expectancy, combined with decreasing birth rates reverses the aging structure of industrialized countries with implications yet unforeseeable. Concomitantly, the probability to suffer from age-related disorders rises dramatically, indicating an urgent need for increasing efforts towards a more comprehensive understanding of the different facets of aging [1], [2].

Given this scenario, the preservation of every-day life competence of aged populations becomes increasingly important. In particular, maintaining of sensorimotor abilities is a crucial prerequisite for a largely independent life. In this context it is important to know whether age-related changes are due to the accumulation of degenerational processes and are thus largely irreversible, or whether they reflect plastic, adaptational and presumably compensatory changes.

Aging comprises a number of physiological modifications, including structural and metabolic changes. While there is a growing body of information about age-related changes at cellular and biochemical levels, and about a decline of cognitive processes such as memory functions, little is known about the effects of aging upon intermediate levels of sensory cortical processing, i.e. the way in which neurons process and integrate information from the external environments.

Imaging studies in aged humans using magnetic resonance imaging to map gray matter density indicated that the time course of aging effects varies considerably over the cortex [3] Similarly, data from longitudinal measures in the regional brain volumes in healthy adults revealed substantial shrinkage of the caudate, the cerebellum, the hippocampus and the association cortices, with minimal change in the entorhinal and none in the primary visual cortex [4].

Aged rats are a convenient animal model in geriatric research as they reach old age within 2 to 3 years. Behaviorally, old rats show a characteristic decline of their sensorimotor state, which consists of a substantial walking impairment [5], [6], [7], [8], [9]. At a cortical level, we have demonstrated that the functional organization of the hindpaw representation in somatosensory cortex of aged rats undergoes a significant deterioration: Receptive fields (RFs) of neurons recorded in the hindpaw representations of aged rats were enlarged, overlap between RFs was increased, and the topographic organization of cortical representational maps was broken down [10].

Here we extend this study by comparing the development of aging effects on the walking pattern with parallel changes of cortical RFs and response latencies of neurons recorded in the fore- and hindpaw representations of somatosensory cortex in the same animal. By that we address the question of how behavioral and cortical changes during aging co-develop. Moreover, comparing fore- and hindpaws allows us to draw conclusions about the global and regional nature of these changes. When comparing individually the cutaneous representations of the fore- and the hindpaw, each animal can serve as its own control. In case of global degenerational processes one would expect rather similar changes to occur in the fore- and the hindpaw representations, which are cortically only a millimeter apart. Furthermore, the comparison between RFs and response latencies allows analyzing whether different parameter changes co-vary, thereby addressing the question of a possible parameter-specificity of age-related changes. Finally, to obtain information about the time course of age-related changes of somatosensory cortex, we used multiple time points. Based on a large numbers of animals of different ages we describe the time course of age-related changes in rat somatosensory cortex, which reveals a substantial regional and parameter specificity indicating that aging processes act rather selectively on cortical processing of sensory information.

## Methods

### Animals

The effects of aging were investigated in a total of 90 male rats. As a baseline, we studied 44 animals aged between 3 and 11 months, denoted as young in the following. These were compared to 43 animals aged between 24 and 39 months of age denoted as old. Additionally we recorded data from 3 rats aged 18, 19 and 22 months; the data of these rats (as an intermediate age group) were included only in the analysis of the time course of cortical changes. All aged animals were hybrid Fischer 344×Brown Norway rats (FBNF1). The young rats were either FBNF1 or Wistar rats. All animals were housed under standard conditions (4 animals/cage in wire topped plastic cages sized 54 cm×38 cm×19 cm) in a 12 h light/12 h dark cycle. Animals had free access to corn-based foodpellets (Hoeveler, 10630) which were kept in a container in the front of the cage. For feeding, the animals had to manipulate the pellets with forepaws and teeth. Water was provided ad libitum through water bottles.

The 50% probability of survival in an aging colony is about 34.5 months for male FBNF1 rats [11], [12] For the age groups studied, gain in body weight during aging in old FBNF1 rats was small: 400 to 450 grams in young vs. 500 to 530 grams in old animals. In addition, the glabrous skin areas of the fore- and hindpaws were not different across age-groups.

### Assessment of walking behavior

**Figure 1:**
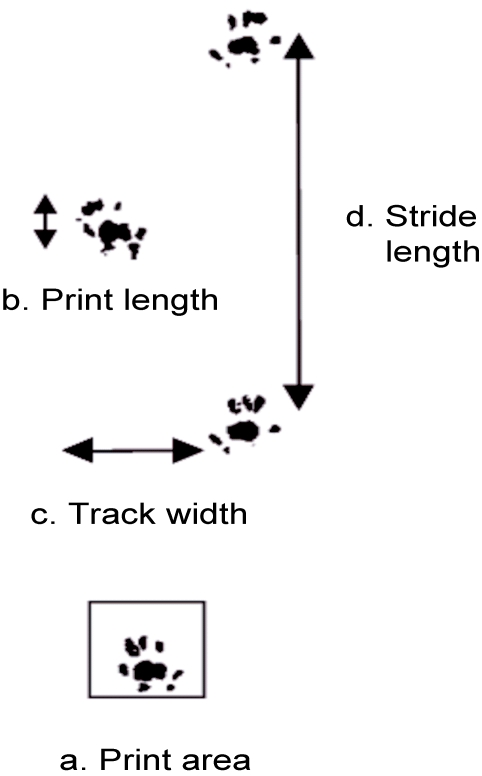
Footprint analysis. Description of the parameters used in the footprint analyses (labeled a–d). a: area of the print (calculated by counting the colored pixels within a frame drawn by the user). b: lengths of the entire print. c: track widths: distance between left and right paw. d: stride lengths, distance between one print and the corresponding following one. Print area and lengths of the prints can be used to characterize limb-specific features of walking, track widths and stride lengths describe global walking properties.

To assess the walking pattern, we obtained footprints the day before the electrophysiological experiments from a total of 18 animals (8 young rats (4 months) and 10 old rats (30 months). The glabrous skin of the forepaws (FP) and the hindpaws (HP)was painted with food coloring. The animals were then allowed to walk through a long small corridor with acrylic walls. The floor was covered with white paper and had an upward slope of 10% to force the animals to walk up an incline to reach a small hutch at the end of the ramp. Behavioral consequences of dysfunctions of neurological origin or of aging on walking abilities can be reliably assessed by analyzing footprint patterns [6], [9], [13]. Here we used footprint recordings as a tool to visualize and quantify age-related changes of walking. The prints were scanned and stored as bitmaps for further analyses with a commercialized software (Footprints 1.21, K. Klapdor & B. Dulfer ©). To characterize the walking patterns of young and old rats we used the following gait parameters: print lengths, surface area of the print (number of colored pixel), stride lengths and track widths (Fig. 1).

### Animal preparation and anesthesia

Animals were anaesthetized with an initial dose of 1.5 mg/g body weight Urethane (Sigma, 20% in water, i. p.). Additional anesthetic (1/8 of the initial quantum) was administered when eye-blinking or paw-withdrawal reflexes could be elicited. Treatment of all animals was within the guidelines of the National Institution of Health Guide and Care for Use of Laboratory Animals (Revised 1987), all experiments were approved by the German Animal Care and Use Committee. The cisterna magna was drained to prevent swelling of the cortex. After a unilateral craniotomy over the paw representations of primary somatosensory cortex and resection of the dura, the cortex was covered with warm silicone oil (DC 200 50cst, Serva). Rectal temperature was kept at 37°C using a feedback-controlled heating pad. The ECG and respiration rate were monitored and were stable throughout every individual experiment.

### Electrophysiology

Glass micropipettes with a tip diameter of 10 µm (OD) broken under microscopic view and filled with 3 M NaCl (2 MOhm at 10 kHz), and a low impedance reference electrode fixed to the neck muscles, were used for recording. Penetrations were usually placed 150 to 250 µm apart, which allowed an accurate definition of the spatial extent of the paw representations. A total of 1549 penetrations (873 in the young rats, of which 600 were in the HP-representation and 273 were in the FP-representation; and 676 in the old rats, of which 310 were in the HP-representation and 366 were in the FP-representation) were made perpendicular to the cortical surface of the primary somatosensory cortex using a motor micro-drive (1 µm resolution) to advance the electrodes. In most cases, multi-unit activity consisting of a small number of action potentials clearly above background was recorded at depths of about 700 µm (layer IV). Penetrations were marked in digitized pictures of the cortical surface with vessels used as landmarks.

### RF measurement and assessment of response latencies

To define the size of cuteaneous RFs on the glabrous skin of the hindpaw we used two different methods. First we used the handplotting technique [14], where RFs are defined as those areas on the skin at which a just visible skin indentation by means of a small probe with a nodular tip of 1 mm diameter evoked a reliable neuronal discharge. Other studies have shown that just-visible indentation is in the range of 250 to 500 µm, which is in the middle of the dynamic range of cutaneous mechanoreceptors [15]. Cells responding either to high threshold stimuli, joint movements or deep inputs were classified as non-cutaneous and were excluded from further evaluation. The location and the extent of RFs were then transferred to a schematic drawing of the paw. RF size was analyzed by calculating the skin area in mm^2^ by planimetry.

**Figure 2:**
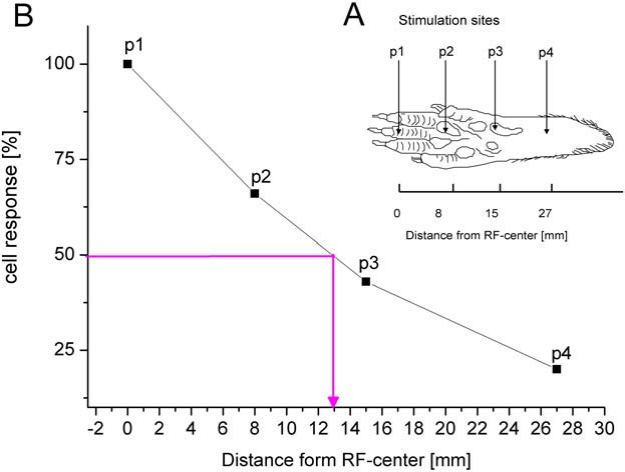
Calculation of RF-length. (A) We used a fixed set of 4 electromagnetic stimulators positioned along the proximal-distal axis of the hindpaw. The first one was positioned on a digit (p1), stimulators 2 and 3 were positioned on the pads and the 4^th^ stimulator was on the more proximal portion of the hindpaw (p4). Tactile stimulation was carried out one after the other at each position. Only RFs were analyzed whose RF were according to hand-plotting localized on a digit. (B) The neuronal response after stimulation at position 1 was set as 100%. The neuronal responses obtained from stimulation at the other positions were normalized and plotted versus the distance from the RF-center. The RF-length was obtained for that distance where neuronal response reached 50% (pink line).

In case of the hindpaw, we additionally used another method which was based on recording action potentials after tactile stimulation using electromagnetic stimulators to obtain an objective, activity-based RF-profile (Fig. 2). Only those RFs were analyzed that, according to hand-plotting, were localized on a digit. For RF-length determination we used a fixed set of 4 stimulators. The first one was located on a digit. The other 3 stimulators were then positioned at selected locations 8, 17 and 27 mm from position 1 along the longitudinal axis of the paw. Tactile stimulation was carried out separately at each position. Stimulus duration was 8 ms delivered at 1 Hz and averaged over 32 repetitions. Then the neuronal response after stimulation at the RF-center was set as 100%. The neuronal responses obtained from stimulation at the other positions were normalized accordingly. The normalized cell responses were plotted versus the distance from RF-center. The criterion for RF-length was set at 50% neuronal response. So for each RF the length could be read off (Fig. 2).

After discrimination of action potentials according to amplitude, they were transformed into TTL pulses and stored in a laboratory PC with a temporal resolution of 1 ms. Response latencies defined as the time difference between stimulus onset and peak response were measured by means of post-stimulus-time histograms (PSTHs) of 1 ms binwidth. Spike acquisition and tactile stimulation was controlled by a Master 8 (AMPI) stimulus generator. Single tactile stimuli could be applied by means of an electromagnetic stimulator, the diameter of the probe was 2 mm. Stimulus duration was 8 ms delivered at 1 Hz at the center of a RF. Neural activity was averaged over 32 repetitions.

All behavioral and electrophysiological data were statistically analyzed using an unpaired, 2-tailed Student's t-test.

## Results

### Age-related changes of walking

**Figure 3:**
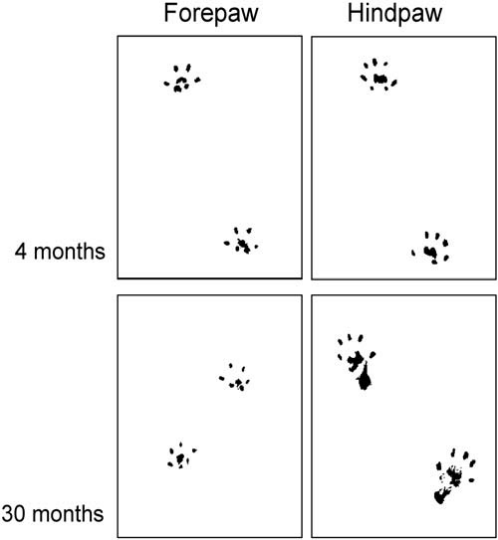
Example of footprints of fore- and hindpaws of young and old rats. The upper panel shows typical prints of fore- (left) and hindpaws (right) of a young rat (4months). Presumably, such prints result from a walking pattern, where only the distal parts of the paw contact the ground. The lower panel shows prints of fore- and hindpaws of an old rat (30 months). The prints of the forepaws (left side) are comparable in young and old animals. In contrast, the prints of the hindpaw of the old animals are considerably larger than those of the young animal indicative of limb-specific effects of aging on walking behavior. Such prints result from a walking behavior where also more distal parts of the paws and the heels are placed on the ground due to reduced muscle forces.

**Figure 4:**
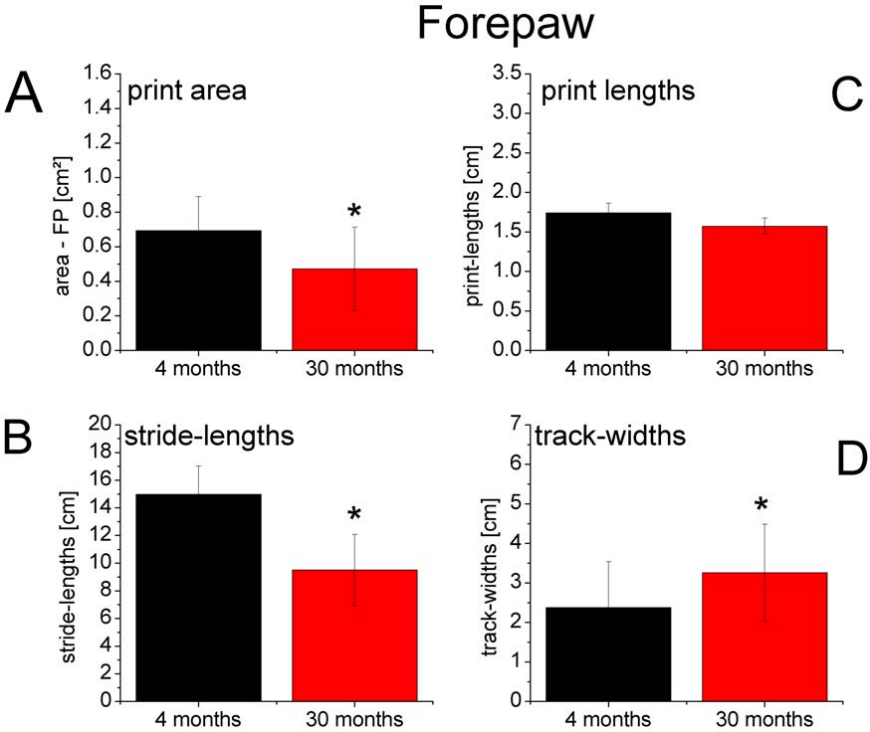
Footprint characteristics of the forepaw. Mean values±SD of the gait parameters print area (A), lengths of the print (C), stride-lengths (B) and track-widths (D) for young (black) and aged rats (red), * p<0.01. (A+C): The limb-specific parameter print area was reduced and print lengths remained unchanged in aged rats, which was not the case for the hindpaw (Fig. 5). (B+D): The global walking parameters stride-lengths decreased and track-widths increased in aged rats. This was also observed for the hindpaw (Fig. 5).

**Figure 5:**
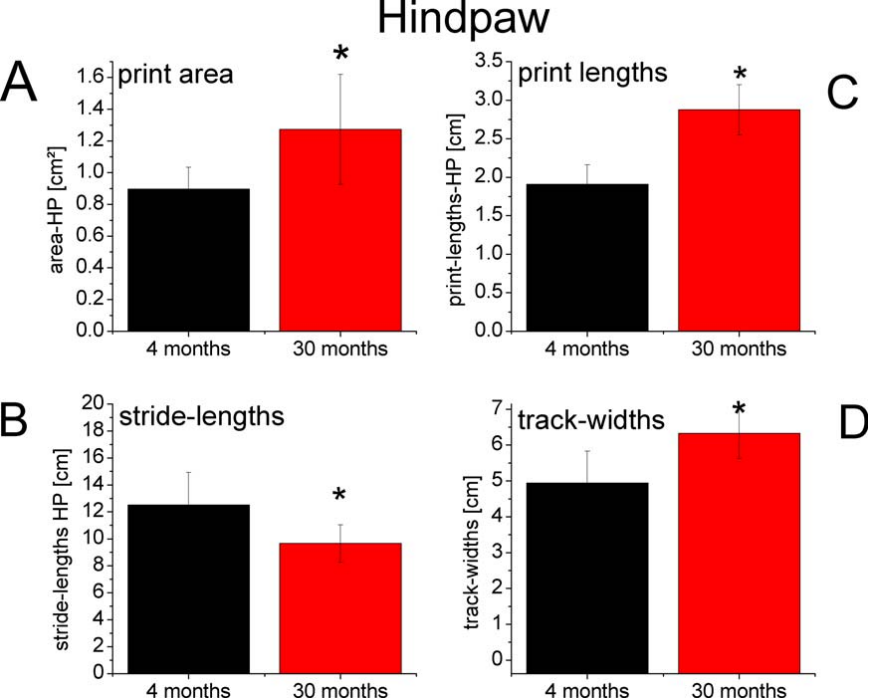
Footprint characteristics of the hindpaw. Mean values±SD of the gait parameters print area (A), lengths of the print (C), stride-lengths (B) and track-widths (D) for the young (black) and the aged rats (red), * p<0.01. (A+C): In contrast to the prints obtained from the forepaws (Fig. 4), the limb-specific parameters print area and the lengths of the prints of the hindpaw were significantly increased in old animals. (B+D): As in the forepaw (Fig. 4), for the global parameters we found decreasing stride-lengths and increasing track-widths for the hindpaw in the aged animals.

Walking behavior and pattern of locomotion were analyzed on the basis of footprint recordings in a total of 18 animals from 2 age groups. Forepaw: 7 young (4 months) and 7 old (30 months) rats; Hindpaw: 8 young (4 months) and 10 old (30 months) rats. Examples of typical footprints taken from fore- and hindpaws of a young and an old animal are illustrated in Fig. 3. The walking pattern of young adult animals was characterized by prints where only the distal aspects of the digits, palms and pads were marked, indicative of a powerful way of moving without engaging the more proximal parts of the paws. In animals of advanced age (30 months) clear age-related changes became apparent, in which the enlargement of the print area of the hindpaws was the most prominent one. In general we observed 2 different types of age related walking changes. The first type we refer to as global alterations was observed in the prints of both the fore- and hindpaws (Fig. 4 and Fig. 5) and consisted of shortening of stride lengths (FP: 14.97 cm vs. 9.50 cm, p<0.01; HP: 12.52 cm vs. 9.66 cm, p<0.01) and an increase of track widths (Figs. 4B, 4D and 5B,5D) (FP: 2.37 cm vs. 3.25 cm, p<0.01; HP: 4.94 cm vs. 6.33 cm , p<0.01). In addition, there were changes in walking that we denoted as local, or paw specific alterations in walking behavior: For the hindpaws, the print areas enlarged (0.89 cm^2^ vs. 1.27 cm^2^, p<0.01) but became reduced for the forepaws (0.69 cm^2^ vs. 0.47 cm^2^, p<0.01), and the print lengths became larger for the hindpaw (1.90 cm vs. 2.87 cm, p<0.01) but remained almost unaffected by age for the forepaw (1.74 cm vs. 1.65 cm, p≤0.08).

### Age-related changes of somatosensory cortical neurons

#### Receptive fields

**Figure 6:**
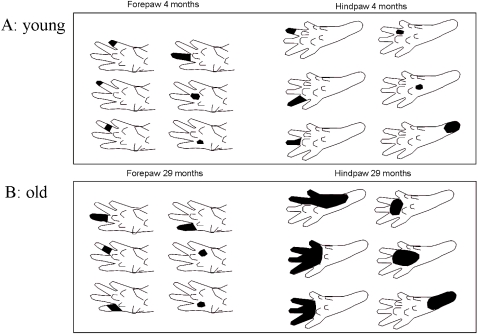
Cutaneous receptive fields (RFs) of the fore- and hindpaw. Typical examples of cutaneous receptive fields (RFs) of neurons recorded in the fore- and hindpaw representation of a rat aged 4 months (A, upper panel) and a rat aged 30 months (B, lower panel). (A) RFs on the forepaw of young rats were very small (left) usually comprising only small parts of a digit or a single digit or pad. RFs on the hindpaw of young rats (right) were slightly larger than on the forepaw, typically consisting of a single digit or parts of a digit. On the more proximal area of the hindpaw, RFs comprised single pads and larger skin areas in the range of the heel. (B) RFs on the forepaw of old rats (left) were only slightly enlarged as compared to young animals. Typical RFs comprised parts of a digit or a single digit or pad. In contrast to the forepaw, RFs located on the hindpaw (right) of old rats were severalfold enlarged. RFs in old rats were characterized by representations of multiple digits and pads and by substantially enlarged RFs in the proximal parts of the paw.

**Figure 7:**
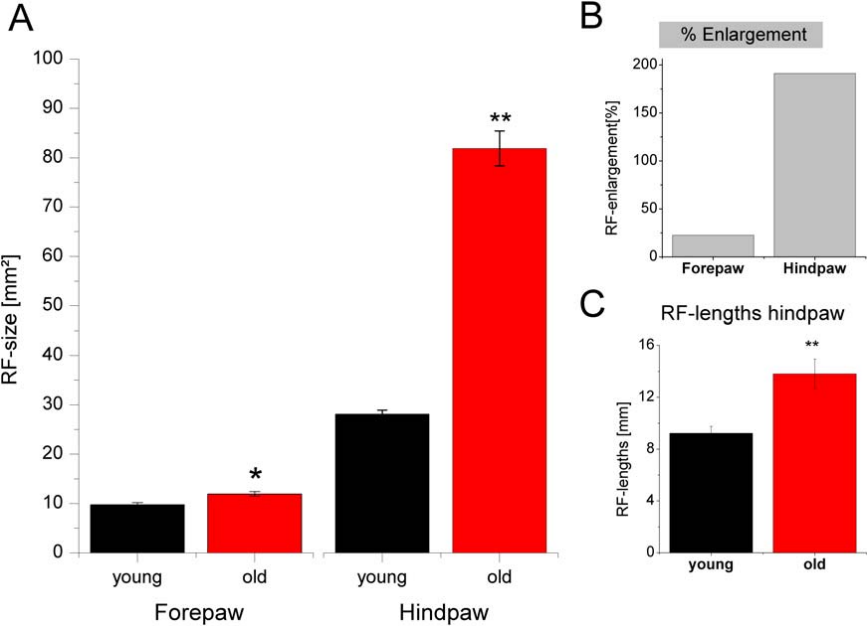
Average RF-sizes of young and old rats. (A) Average RF-sizes of the fore- and hindpaw of young (black) and aged (red) animals are shown. Error bars indicate SEM. * p<0.01, ** p<0.001. (B) Normalized age-related RF-enlargement. The increase of RF-size was 22% for the forepaw and 190% for the hindpaw. Although we found that RFs were enlarged on both the fore- and the hindpaw of old animals, the effects of aging on the RF-size was substantially stronger on the hindpaw than on the forepaw. (C) Mean values (±SEM) of RF-length. ** p<0.001. RF-lengths was only measured for hindpaw RFs of young (black) and old (red) rats. Analogous to the RF-size determined by handplotting we found a significant increase of RF-lengths for the old animals.

To illustrate the overall effects of aging on cortical RFs recorded in the fore- or hindpaw representation of primary somatosensory cortex, Fig. 6 shows typical examples found in a young adult (4 months, Fig. 6A), and in an old animal (29 months, Fig. 6B). In young animals, RFs located on the forepaw usually comprised only parts of a single digit, or single pads. Similarly, in old rats most forepaw RFs covered only parts of a digit even though slightly enlarged. RFs on more distal parts of the forepaw usually represented single pads. In case of the hindpaw, the characteristic RFs of a young rat comprised only single digits, parts of a single digit or parts of two neighboring digits. RFs located in the medium portion of the paw usually represented single pads. RFs of the proximal part of the paw normally comprised slightly larger skin areas than the more distal RFs. In contrast to the moderate (about 22%, Fig. 7A) but significant (t-test, p≤0.01) enlargement of forepaw RFs (Fig. 7), a severalfold increase of RF size was observed for the hindpaw of aged rats. Hindpaw RFs in aged animals were characterized by representations of multiple digits and pads and by substantially enlarged RFs in the proximal parts of the paw (Fig. 6B) resulting in a significant (p≤0.01) overall increase of RF size compared to adults by 190% (Fig. 7A). The mean RF-size on the forepaw of the young rats was 9.74 mm^2^, the averaged RF-size of the old rats was 11.94 mm^2^. In case of the hindpaw the mean value for the young animals was 28.15 mm^2^, the mean value of the old rats was 81.89 mm^2^.

In the case of the hindpaw RFs we additionally measured RF-profiles based on recordings of PSTHs at 4 defined locations along the proximal-distal axis of the hindpaw. As a measure of RF length we calculated that distance from the RF-center where neuronal responses in the PSTHs reached 50% of the maximal activity in the RF-center (RF-length). Average RF-lengths also differed significantly between young and old animals (Fig. 7B), p<0.01). We found 9.21 mm+/−0.52 for the young animals and 13.80 mm+/−1.14 for the aged animals. RF-size values obtained with both methods showed a high correspondence (Pearson's linear correlation r = 0.77, p<0.0001) confirming the data obtained from handplotting. This comparison indicated that handplotting provides reliable and reproducible information about RF size and location given that stable and fixed criteria are used.

#### Response latencies

**Figure 8:**
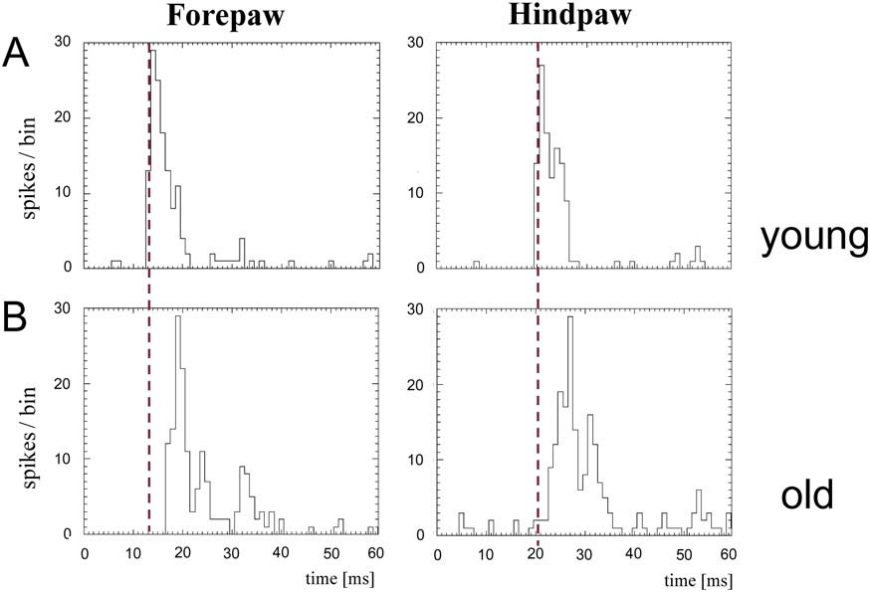
Response latencies of cortical neurons recorded in the fore- and hindpaw map. Examples of neuronal responses in form of post-stimulus time histograms (PSTHs) after tactile stimulation applied to the center of a RF. The number of spikes/bin (binwidth = 1 ms) is plotted against time. The red dotted line marks the time of maximal cell response (peak latency) in the PSTHs of fore- (left) and hindpaw (right) neurons of the young animal (upper panel). (A) Examples of PSTHs recorded in the fore- (left) and in the hindpaw representation (right) of a young rat (5 months). (B) Examples of PSTHs recorded in the fore- (left) and in the hindpaw representation (right) of an old rat (29 months). Response latencies were lengthened in aged animals by approximately the same amount for neurons of both representations.

**Figure 9:**
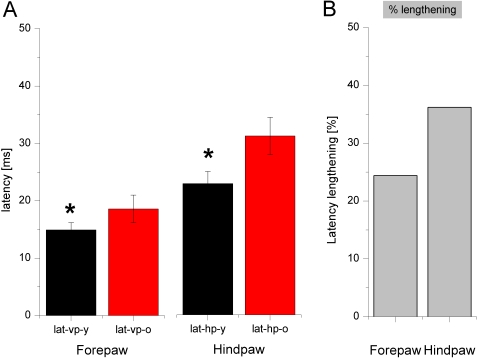
Averaged latencies of young and old rats. (A) Averaged peak response latencies of neurons from fore- and hindpaw-representations of young (black) and aged (red) animals are shown. Error bars indicate the SEM. * p<0.01. (B) Normalized age-related lengthening of peak response latencies. The lengthening was 24.4% for the forepaw and 36.2% for the hindpaw. In contrast to the parameter RF-size the lengthening of latencies was similarly affected in cortical neurons of fore- and hindpaw-representation.

To study possible effects of aging on response latencies following tactile stimulation of the glabrous skin of the paws, we measured peak response latencies in young and aged animals. Response latencies were defined as the time differences between onset of stimulation and maximal peak responses. In Fig. 8, examples of post stimulus time histograms (PSTH) from a young (5 months, Fig. 8A), and an aged animal (29 months, Fig. 8B) are shown. In contrast to the findings observed for RF analysis, in aged animals cortical latencies for both the fore- and the hindpaw were similarly affected. Latencies of neurons from both representations showed significant lengthening with age (Fig. 9). The averaged latencies for neurons in the FP-representation were 14.94 ms±1.6 for the young rats and 18.59 ms±2.4 for the old rats (p≤0.01). For neurons of the HP-representation averaged latencies were 22.98 ms±2.09 for the young animals and 31.20 ms±3.23 for the old animals (p≤0.01). Compared to the young rats this is a lengthening of about 24% in case of the forepaw and about 36% in case of the hindpaw.

#### Time course of cortical changes

**Figure 10:**
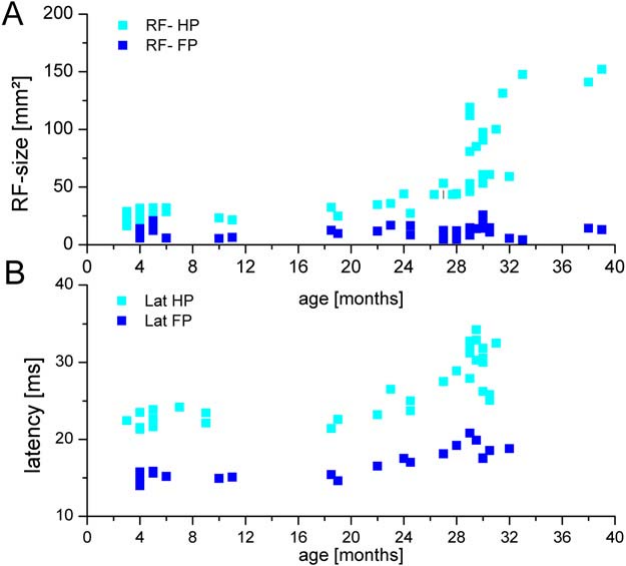
Timecourse of age-related changes. (A) Averaged RF-size for each individual animal plotted as a function of age. RFs on the hindpaw (light blue squares), RFs on the forepaw (dark blue squares). Until an age of approximately 24 months we found no age related alterations of RF-size of fore- and hindpaw cortical neurons. Beyond that age RFs on the hindpaw increasingly enlarged with advancing age, with the largest RFs found in the animals with the highest age. For the forepaw no comparable increase was observed. (B) Averaged peak-latencies for each individual animal plotted as a function of age. Light blue squares Latencies of neurons recorded in the representation of the hindpaw (light blue squares), latencies of neurons from the forepaw representation (dark blue squares). Similar to RF-size, we found no age related lengthening of peak latencies until an age of about 24 months. From then on latencies of neurons from both the fore- and the hindpaw representation were increasingly lengthened with advancing age.

The time course of the age-dependency of the parameters described above is illustrated in Fig. 10. Here we plotted for each individual animal the average values for RF size and latency, separately for the fore- and the hindpaws. The data illustrate that age-related changes of cortical RFs and latencies appear to develop rather late at approximately 24 months of age. Until that age, almost no changes could be detected. More severe alterations are present at an age of about 28 to 29 months. As described above, major changes of RF size are largely limited to the hindpaw, while changes in latencies of fore- and hindpaw develop rather in parallel. Interestingly, even for the animals of most advanced age no changes in forepaw-RF comparable in magnitude to those seen in the hindpaw could be detected.

#### Paw-dependent correlation between RF size and latency

In those animals, where RFs and latencies could be recorded both in the fore- and in the hindpaw representation a closer analysis between co-changes of different parameters and different representations could be performed. Results of a linear correlation analysis (Pearson) are summarized in Table 1. Significant correlations were observed for co-changes of latencies of neurons recorded in the fore- and in the hindpaw, and for co-changes of latency and RF size for neurons of the hindpaw, i.e. lengthening of average latency co-varied for both cortical representations. Similarly, latency lengthening and RF enlargement co-varied within the hindpaw representation. No such correlations could be observed for co-variation between latency and RF size of neurons of the hindpaw representation, nor for co-variation between the enlargements of RF size in both cortical representations.

**Table 1:** Correlation analysis between fore- and hindpaw parameters.

	RF: FP-HP	latency: FP-HP	FP: latency-RF	HP: latency-RF
**r**	0.28421	0.78165	0.06846	0.81966
**p**	0.1033	0.00021	0.76808	5.352E-08
**# animals**	34	17	21	29

## Discussion

Our results demonstrate age-related changes in the functional properties of rat cortical somatosensory neurons representing the fore- and hindpaw. While RFs of hindpaw neurons enlarged severalfold with age, we found only a minor enlargement for RFs of forepaw neurons even in the same individual. In contrast, response latencies of neurons recorded in both the fore- and the hindpaw representation were similarly affected by age. These data suggest that aging affects cortical neurons differentially dependent on the body part they represent. Similarly, behavioral assessment of walking revealed an overall age-related shortening in stride length and track width and, restricted to the hindlimb, an increase of print area and print length indicative of a limb-specific age-related walking impairment.

### Changes of walking behavior in old rats

We found two major types of changes in the footprint pattern of fore- and hindpaw of aged animals. First, the footprint analysis confirmed previous studies [5], [7], [8] about a general impairment of walking in aged rats, which affects fore- and hindpaw similarly. Reduced stride length is also a characteristic feature of walking behavior in elderly human subjects, while increased track width subserves the maintenance of postural stability [16]. In addition, we observed limb-specific alterations such as enlarged print areas and print lengths only for the hindpaw of aged animals, which were not observed in the prints of the forepaw. These changes are most likely caused by a walking behavior where not only the distal parts of the hindpaw, but also the more proximal parts touch the ground. According to unpublished data, extensor muscles of the hindpaw are particularly affected by age resulting in reduced ground reaction forces providing further evidence that the hindlimbs are more severely affected by aging processes (Schulz, M.H., Dinse H.R. unpublished). On the other hand, the parallel development of decreased print areas of the forepaws in old animals might be due to modifications in the placement of the forepaws to compensate for the altered muscular properties of the hindpaws [17], [18], [19].

### Age-related changes: degenerative or use-dependent?

A major finding of our study was the apparent link between cortical and behavioral alterations at high age. We observed a rather uniform age-dependent lengthening of response latencies that co-develop in both the fore- and the hindpaw representation. Response latencies as measured in our experiments consist of several components such as peripheral and central conduction times, synaptic transmission and synaptic integration. Particularly, conduction velocity is known to slow down during aging due to demyelinization [20], [21], [22]. It is therefore conceivable that changes of latencies can be regarded as a signature of global, regionally unspecific age-related changes. Because the hindpaw is further distal to the brain than the forepaw response latencies of the forepaw are systematically shorter. If conduction velocity is slowed down, conceivably its consequences are more pronounced for long-distance transmission as was the case for the hindpaw.

In contrast we found that the magnitude of age-related changes of cortical RFs was not uniform across different representations of the body. While for the cortical region representing the skin areas of the forepaws only small, yet significant enlargements of RFs were observed in aged animals, RFs recorded in the hindpaw representation enlarged almost threefold. It should be emphasized that the magnitudes of age-related alterations of RF size and response latencies cannot be compared directly, as the overall lengthening of latencies and conduction is limited by physiological factors, while increase of RF size is limited by the dimensions of the skin area of the paws, which allows for much higher percentual changes.

We speculate that the continued use of the forepaws in feeding and grooming behavior in animals of high age might be causally related to the observation that the forepaw RFs show less age-related alterations. On the other hand, the modified use of the hindpaw as inferred from the altered foot prints might be causally related to the severalfold enlargement of the RFs of the hindpaw. It is conceivable that touching the ground with larger areas of the hindpaw alters the cutaneous inputs resulting in a temporal and spatial coactivation of almost the entire glabrous skin surface. Coactivation has been shown to be a major driver of cortical plasticity, presumably through Hebbian mechanisms, which causes RFs to enlarge by means of a fusion of single RFs [10], [23], [24], [25].

When comparing the cutaneous representations of the fore- and the hindpaw each individual animal serves as its own control. In case of degeneration one would expect comparable changes to occur in both the fore- and the hindpaw representation. However, analysis of receptive fields in the cortical forepaw representation of animals of high age revealed only little alterations. These results imply that age-related changes can be very region-specific, and they implicate a link between neural changes and specific behavioral alterations emerging during aging. Because of this association, it appears conceivable that aspects of age-related changes reflect plastic reorganization as a consequence of altered use rather than degenerational processes developing during aging. A major prediction arising from this assumption is that age-related changes of this type are not irreversible, but subject to training and stimulation protocols, which is treated below.

### Comparison of tactile perception of human hand and foot

Tactile perception and performance depend on the intactness of cortical substrates subserving the sense of touch. Accordingly, deterioration of cortical somatosenory processing can be expected to affect measures of tactile performance. A recent study on gap detection revealed that with increasing age, substantial differences in the amount of age-related impairment developed on the great toe (about 400% decline in comparison to young subjects), while impairment on the finger tips was only 130% [26]. These results were interpreted by the authors as indication that aging acts non-globally. These data also imply that our results described for aged rats might bear relevance for humans, as they confirm significant differences in the magnitude of age-related perceptual changes of hand and foot.

### Relation to age-related changes of periphery or cell numbers

To show that the described age-related changes of cortical sensory processing are not merely a simple reflection of changes occurring already at the periphery, we have previously investigated the effects of aging on rapidly (RA) and slowly adapting (SA) cutaneous mechanoreceptors by means of single fiber recordings and evoked sensory nerve action potentials (EAPs) of the hindpaw of the N. plantaris in adult and old rats [27]. EAPs revealed comparable shapes and amplitudes in all animals of all age groups. In aged rats, conduction velocities were significantly lengthened by about 15%, and mechanoreceptor composition was different from adults, resulting in a lower number of SA units. However, there were no differences in RF size and in threshold between old and adult animals [27]. In monkey retina, stereological procedures used to compare the densities, numbers, and soma sizes of retinal ganglion cells in young adult and old rhesus monkeys revealed no changes with age [28]. Similarly, single cell recordings in the monkey LGN (lateral geniculate nucleus) analysing a broad spectrum of response properties suggested that effects of aging on the functional properties of LGN neurons are relatively subtle or absent [29] In addition, there appeared to be no significant cell loss in the LGN or striate cortex [28], [30], [31]. On the other hand, at a level of visual cortex, severe age-related alterations of RFs and RF properties had been reported [32], [33].

### Treatability of age-related changes

Our findings and interpretation extend the framework of use-dependent plasticity to high age. It is well documented that in animals and humans intensified sensory stimulation, as enforced through extensive use or training, modifies cortical processing and cortical representations of the respective body parts [34], [35], [36], [37]. On the other hand, studies describing the role of reduced sensory inputs on cortical reorganization reported shrinkage of representational maps [38]. Assuming that aging is linked to neuroplasticity principles a strong prediction can be made: If age-related changes are plastic-adaptive in nature they should be reversible by training or specific stimulation protocols.

In rats, behavioral challenges through an enriched environment have been shown to affect many morphological and physiological parameters including neurogenesis [39], [40], [41], [42], [43]. Interestingly, for aged rats that were kept in enriched conditions for their entire life beneficial effects on cortical forepaw neurons have been reported [44]. We have addressed whether age-related changes can be affected through enriched housing even after they developed. Rats at an age of 26 to 29 months exposed to enriched environments for a few months regained nearly normal walking and sensorimotor behavior of the hindlimbs and the typical age-related enlargement of RFs of the hindpaw was almost eliminated. In contrast, the age-related prolongation of response latencies remained unaffected supporting its degenerational character resisting interventional measures (Churs et al., 1996, Soc Neurosci Abstr. 22:102). These findings indicate the treatability of some but not all age-related changes. Most importantly, they clearly imply that specific age-related changes can be reversed after they have developed, which we take as a convincing argument for their non-degenerative properties.

In humans, there is convincing evidence that age-related changes can be ameliorated through intense schedules of training and practice [45], [46], [47], [48], [49].

Tactile acuity in elderly subjects is severely impaired [26], [50], [51], [52], [53], but impairment is much stronger in sighted than in blind people of the same age [54]. These results show that discrimination can be almost unimpaired even in elderly individuals supporting the hypothesis that practice is a major driving force for maintaining high acuity performance even into old age. As an alternative approach to training we have recently introduced tactile coactivation in order to improve tactile performance in humans. Coactivation closely follows the principles of Hebbian learning and induces improvement of tactile performance in parallel to cortical reorganization [50], [55], [56]. Application of the same protocol in elderly subjects resulted in a substantial amelioration of age-related decline in tactile acuity, demonstrating that age-related decline of perception is not irreversible but can be improved by specific stimulation protocols.

### Conclusion

We show that age-related changes of somatosensory cortical neurons display a complex pattern of regional specificity and parameter-dependence. The fact that, in recordings of the same individual animal, RFs that are only separated by a few millimeters may reveal either severalfold enlargement or only very mild changes argues against degenerational processes on a uniform and global scale as a cause of these changes. Instead, the close associations between behavioral changes that mirror the pattern of age-related cortical changes argue for a scenario where age-related alterations rather reflect a mix of plastic-adaptive changes in response to altered use and degenerational changes developing with age. In this way we can separate and identify parameters of cortical processing that are differentially affected during aging, opening the perspective that plastic adaptive changes are not irreversible but subject to amelioration through training and stimulation.
